# Intrauterine interventions for women with two or more implantation failures: A systematic review and network meta-analysis

**DOI:** 10.3389/fendo.2022.959121

**Published:** 2022-08-29

**Authors:** Xin Hang Jin, Yang Li, Dan Li

**Affiliations:** ^1^ Department of Gynecology, Affiliated Hangzhou First People’s Hospital, Zhejiang University School of Medicine, Hangzhou, China; ^2^ Department of Obstetrics and Gynecology, First Affiliated Hospital, School of Medicine, Zhejiang University, Hangzhou, China; ^3^ Department of Ultrasound, Hangzhou Red Cross Hospital, Hangzhou, China

**Keywords:** intrauterine, PRP, PBMC, endometrial scratch, implantation failure

## Abstract

**Objective:**

To compare the effectiveness of different intrauterine interventions for women with two or more unexplained implantation failures.

**Design:**

A systematic review and network meta-analysis of randomized controlled trials (RCTs).

**Patient(s):**

Women with two or more implantation failures undergoing fresh or frozen embryo transfer (ET).

**Intervention(s):**

An electronic search of the following databases: Pubmed, Cochrane Central Register of Controlled Trials (CENTRAL), and Embase.

**Main Outcome Measure(s):**

Clinical pregnancy, live birth/ongoing pregnancy, and miscarriage.

**Result(s):**

We included 21 RCTs(3079 women) in the network meta-analysis. The network meta-analysis showed that compared with control treatment, platelet-rich plasma(PRP), peripheral blood mononuclear cells (PBMC), granulocyte colony-stimulating factor(G-CSF), human chorionic gonadotropin(HCG), and endometrial scratch(ES) significantly increased clinical pregnancy(OR 3.78, 95% CI 2.72 to 5.25; 2.79, 95% CI 1.75 to 4.45; 1.93, 95% CI 1.37 to 2.72; 1.80, 95% CI 1.18 to 2.72; 1.75, 95% CI 1.29 to 2.36, respectively). PRP ranked the highest in improving clinical pregnancy, followed by PBMC, G-CSF, HCG, and ES. Compared with control treatment, PRP, PBMC, and ES significantly increased live birth/ongoing pregnancy (OR 5.96, 95% CI 3.38 to 10.52; OR 2.55, 95% CI 1.27 to 5.11; OR 1.70, 95% CI 1.07 to 2.69, respectively). PRP ranked the highest in improving live birth/ongoing pregnancy, followed by PBMC, and ES.

**Conclusion(s):**

PRP is the most effective intrauterine intervention in improving pregnancy outcome in women with two or more implantation failures.

## Introduction

Infertility remains a major issue in human reproduction, affecting as many as 186 million people globally (1). In developed countries, approximately 9% of the population suffers from this condition, and more than 56% of the couples seek advice for assisted reproduction treatments (2). Although great advances in assisted reproduction techniques(ART) have been achieved over the past few decades, the success rate of *in vitro* fertilization(IVF) is still relatively low; the clinical pregnancy and live birth rates per embryo transfer(ET) range between 30%-40% and 20%-30%, respectively (3, 4). Embryo implantation remains the major obstacle for the success of IVF or intracytoplasmic sperm injection(ICSI) cycle, and it has been estimated that 70% of pregnancy loss is due to implantation failure (5). Furthermore, about 10% women undergoing IVF cycle suffer from recurrent implantation failure(RIF) (6). RIF is defined as the repeated transfer of a good-quality embryo to a healthy uterus without achieving successful implantation and pregnancy (7). However, there is no consensus on the number of failed cycles and good-quality embryos needed to define RIF, and different IVF centers may use different definitions for RIF (8–11). The number of previous failed cycles may range from 2 to 6, and the number of previously transferred embryos may range from 3 to 10 or more (6, 12). Although the potential etiologies of RIF are diverse, embryo quality and intrauterine environment play a major role in the success of implantation (8, 13, 14). However, implantation may still fail during an IVF-ET cycle even after the transfer of good-quality embryos, which indicates intrauterine environment is crucial for successful implantation. RIF imposes a significant psychological and financial burden on infertile couples and remains a challenging to clinicians. Various intrauterine interventions have been proposed to facilitate embryo implantation in women with implantation failure by improving endometrial receptivity (15). These interventions include endometrial scratch(ES), intrauterine perfusion of human chorionic gonadotropin(HCG), granulocyte colony-stimulating factor(G-CSF), autologous peripheral blood mononuclear cells (PBMC), and autologous platelet-rich plasma(PRP). ES is a procedure that involves mechanical endometrial injury with the use of a pipelle or similar sampling device with the intention to improve endometrial receptivity. HCG, which is the homologous isomer of LH, can regulate both endometrial receptivity and embryo implantation by inducing the secretion of various cytokines in endometrium during the implantation window (16). G-CSF, which is a multi-potential cytokine produced by monocytes, macrophages, fibroblasts, endothelial cells, and bone marrow stromal cells, has specific receptor on various tissues especially on trophoblast and endometrial cells and can modulate the function of neutrophil and influence cytokine release (17–21). Autologous cultured PBMC, which consists of T lymphocytes, monocytes, and B lymphocytes, can modulate the production of several cytokines and promote embryo implantation as well as endometrial receptivity (22). PRP is autologous plasma that is obtained by sequestering and concentrating platelets from fresh whole blood, contains a high concentration of platelet 4-5 times above the normal range and has pro-regenerative properties (23). Recent studies have shown promising results for these interventions in women with implantation failures. However, it is unclear that which is the most effective intrauterine intervention in improving pregnancy outcome in women with two or more implantation failures.

## Materials and methods

### Search strategy and selection criteria

We conducted and reported our study in accordance with the preferred reporting items for systematic reviews and meta-analyses (PRISMA) extension statement for network meta-analysis (24). We carried out an extensive electronic search for publications without language restrictions in the following databases: the Cochrane Central Register of Controlled Trials (CENTRAL), PubMed, and Embase. The following key words, MeSH terms and their combinations were used in our search strategies: endometrial injury; endometrial scratch; endometrial biopsy; endometrial sampling; endometrial damage; granulocyte colony stimulating factor; G-CSF; human chorionic gonadotropin; HCG; peripheral blood mononuclear cell; PBMC; platelet rich plasma; PRP; assisted reproductive techniques; *in vitro* fertilization; intracytoplasmatic sperm injection; ICSI; implantation failure; embryo implantation; and embryo transfer. Appropriate suffixes were used for each database. We also manually searched the reference lists of the initially identified articles, previously published reviews for additional relevant publications. We performed the most recent search on May 1, 2022.

We included studies comparing one or more treatments with placebo, with no treatment, or with each other for women with two or more implantation failures. Studies were included if they met the following criteria: 1. The studies were randomized controlled trials(RCTs), and other studies including quasi-RCT, cohort studies or case-control were excluded. 2. The treatments were various intrauterine interventions. 3. the control was any other intrauterine intervention, placebo or no intervention. 4. the participants were women with two or more implantation failures undergoing fresh or frozen ET. 5. Outcome measures were pregnancy outcomes including clinical pregnancy, live birth, or miscarriage.

### Data extraction and assessment of risk of bias

Two reviewers(Y.L.,D.L) independently screened the titles and abstracts to identify potentially eligible trials and then retrieved and assessed the full texts of the relevant citations for inclusion. We extracted data from included studies on population characteristics, study design, sample sizes, intervention details and reported outcomes. Any discrepancies between the two reviewers(Y.L.,D.L.) were solved unanimously through discussion.

The primary outcome was clinical pregnancy. Clinical pregnancy was defined as gestational sac or fetal heartbeat observed on ultrasound. Secondary outcomes included live birth/ongoing pregnancy, and miscarriage. In case live birth was not reported, we used ongoing pregnancy.

We assessed the methodological quality of the included trials using the Cochrane risk of bias tool (25). This tool evaluates seven domains of risk of bias (random sequence generation, allocation concealment, blinding of participants and personnel, blinding of outcome assessment, incomplete outcome data, selective reporting and other bias). The authors’ judgments were expressed as “low risk”, “unclear risk” or “high risk” of bias for each domain.

### Data synthesis and statistical analysis

A network meta-analysis was conducted to simultaneously compare various intrauterine treatments with placebo, with no treatment, or with each other for each outcome. Whenever possible, statistical analyses were carried out with an intention to treat approach(number of events per women randomized). Network plots were constructed to illustrate the geometry of the network. Placebo, and no treatment were considered as the same node in network meta-analysis, and various endometrial biopsy and sampling were considered as ES. All network meta-analyses were performed with a random effects multivariate meta-analysis model in Stata software (version. 14.0, Stata Corp.). For network meta-analysis, summary treatment effects were expressed as odds ratios(ORs) with a corresponding 95% confidence intervals (CIs). We used the surface under the cumulative ranking curve(SUCRA) to rank the treatments. The SUCRA was used to provide a hierarchical ranking of the different treatments. The efficacy of every interventions, expressed as a percentage, was considered in relation to an imaginary intervention assumed to be the best. Higher SUCRA values therefore correspond to more effective treatments.

## Results

### Literature search results

**Figure 1:**
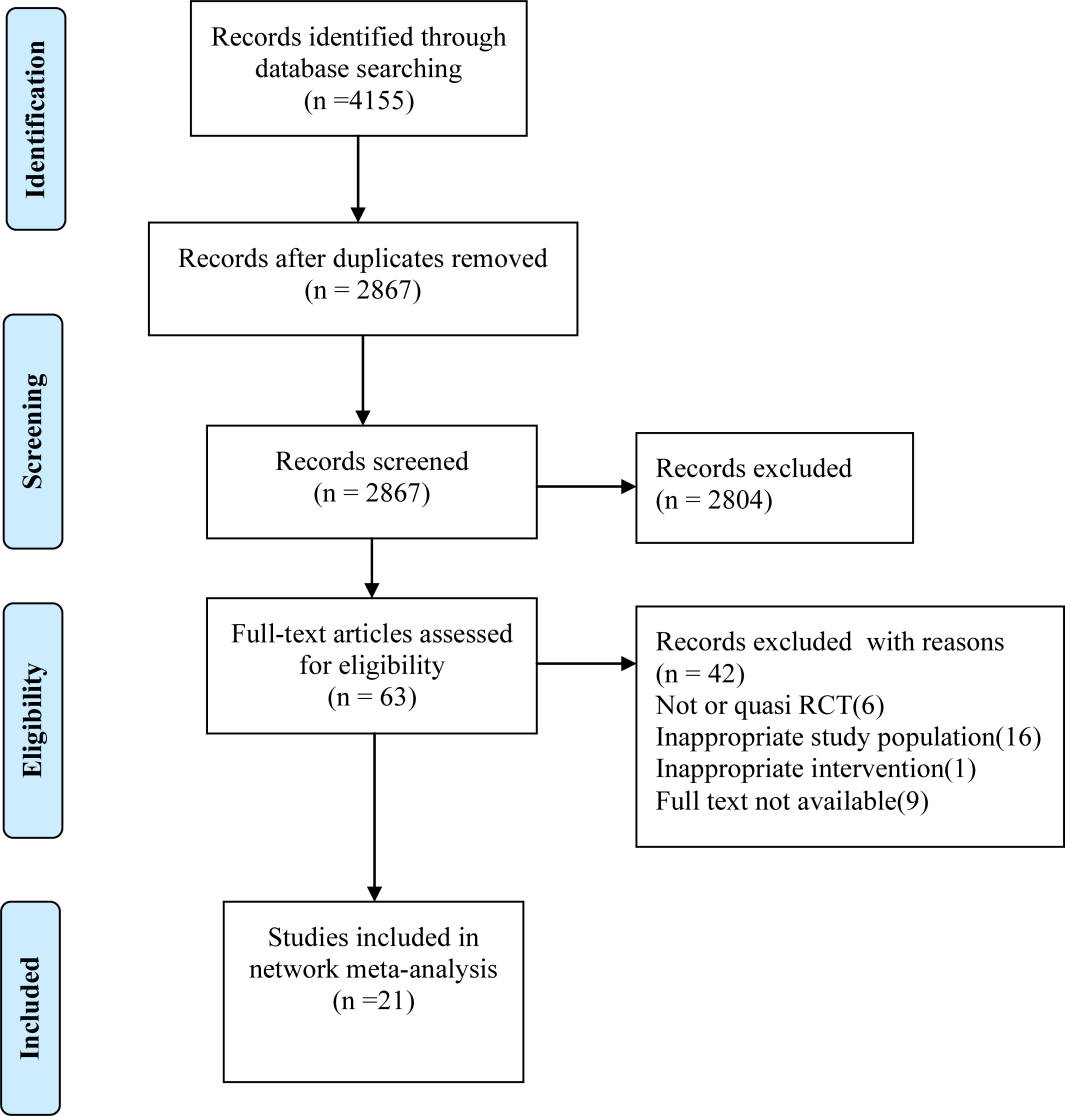
PRISMA flow diagram of study selection.

Our literature search identified 4155 citations, of which, 1288 were duplicates, and 2804 were excluded based on title and abstract. After assessing full texts of the 63 potentially eligible citations, 21 RCTs(3079 women) were included in the present systematic review and network meta-analysis. The PRISMA flow diagram illustrating the selection procedure is presented in **Figure 1**.

### Characteristics of the included studies

The characteristics of these included studies are shown in **Supplemental Table S1**. Twenty studies were written in English, and one study was written in Chinese. Twenty studies was reported as full-text publications, while the remaining one study was reported in conference abstract. The publication dates of the included studies ranged from 2009 to 2022. Most of the studies were performed in Asia, while few studies were conducted in Europe. Seventeen studies were single center RCT, two studies was double-centered, and only study was multiple center RCT. The 21 RCTs included 3079 women, of which 1514 were allocated to the intervention group, and 1565 to the control group. The mean age across studies ranged from 21 to 45 years, and the mean number of previous embryo transfer failures varied among studies. Nine studies enrolled women with two or more previous implantation failures, whereas 12 studies included women with three or more previous implantation failures. Most of the studies included women with previous good-quality embryo or blastocyst transfer failures. All the included RCTs compared at least two of the 6 treatments: ES, G-CSF, HCG, PBMC, PRP, and placebo. Of these 21 studies, 3 studies had three comparisons: one study compared two interventions with control treatment; one study used the same intervention of two different routes of injection as treatments; one study used two different placebo treatments as control groups. The remaining 18 RCTs had two comparisons(treatment vs control). Pituitary block was achieved by using GnRH-agonist long protocol, GnRH-agonist short protocol, or GnRH-antagonists scheme. Fresh ET was performed in 10 studies, frozen ET was performed in 10 studies, and one study performed fresh or frozen ET.

### Risk of bias assessment results

All the studies but four used adequate methods of random sequence generation. Only 7 studies that described allocation concealment were judged “at low risk of bias”, whereas the remaining studies were judged “at unclear risk of bias”. The results of the risk of bias assessment are shown in **Supplemental Figure S1**.

### Network meta -analysis results

#### Primary outcome: Clinical pregnancy

**Figure 2:**
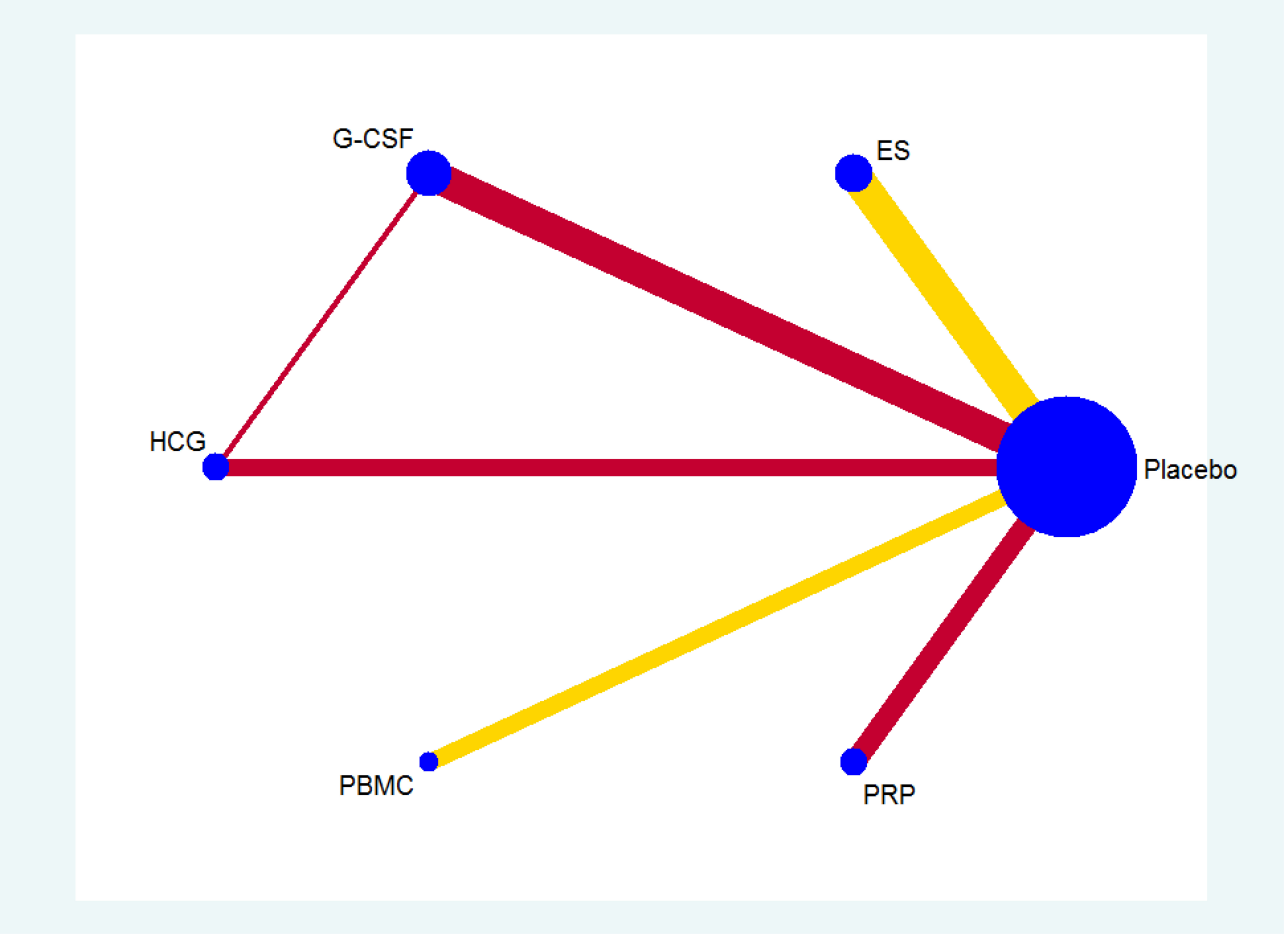
Network plot for clinical pregnancy.

**Figure 3:**
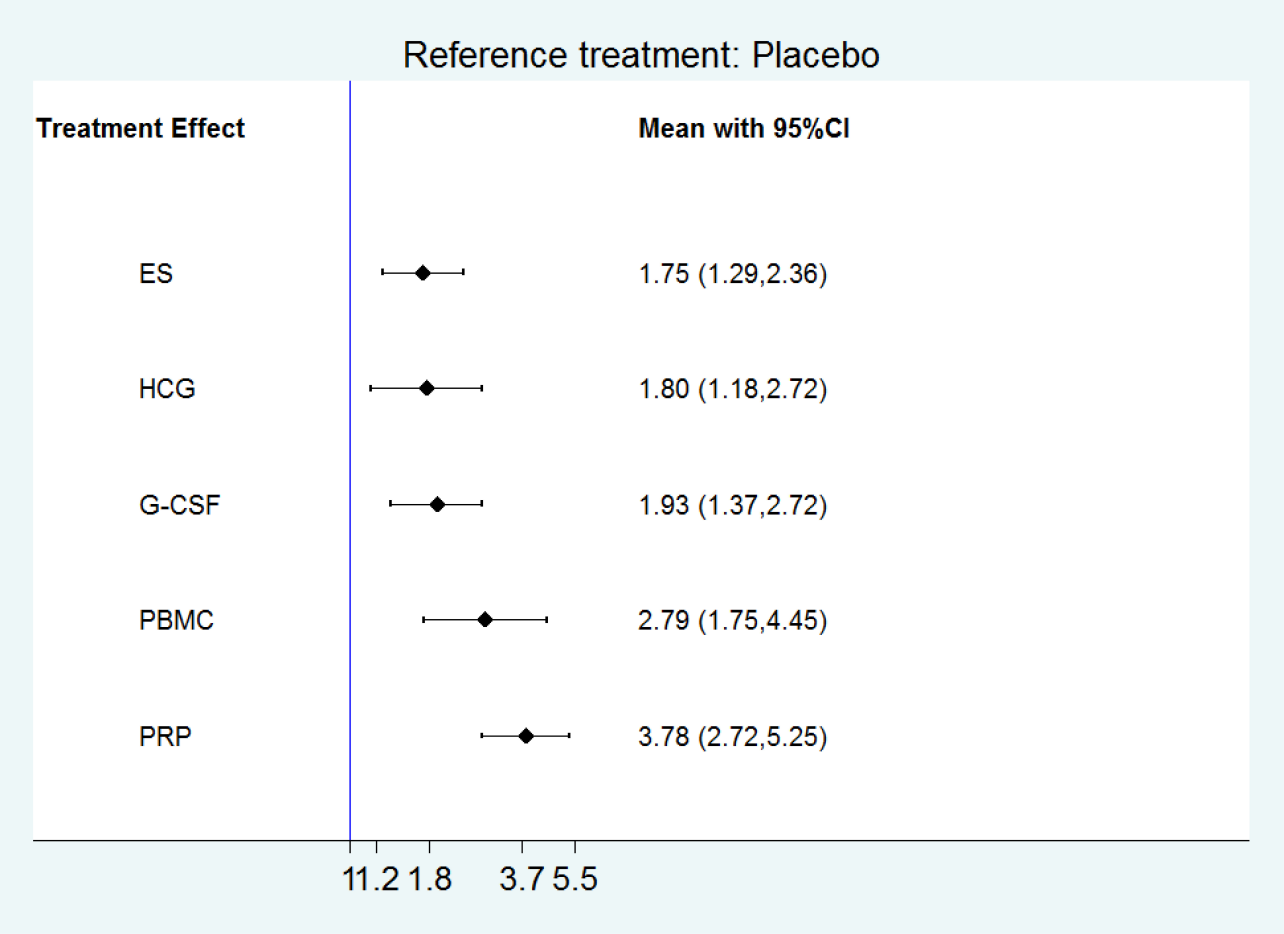
The results of the network meta-analysis for clinical pregnancy.

**Figure 4:**
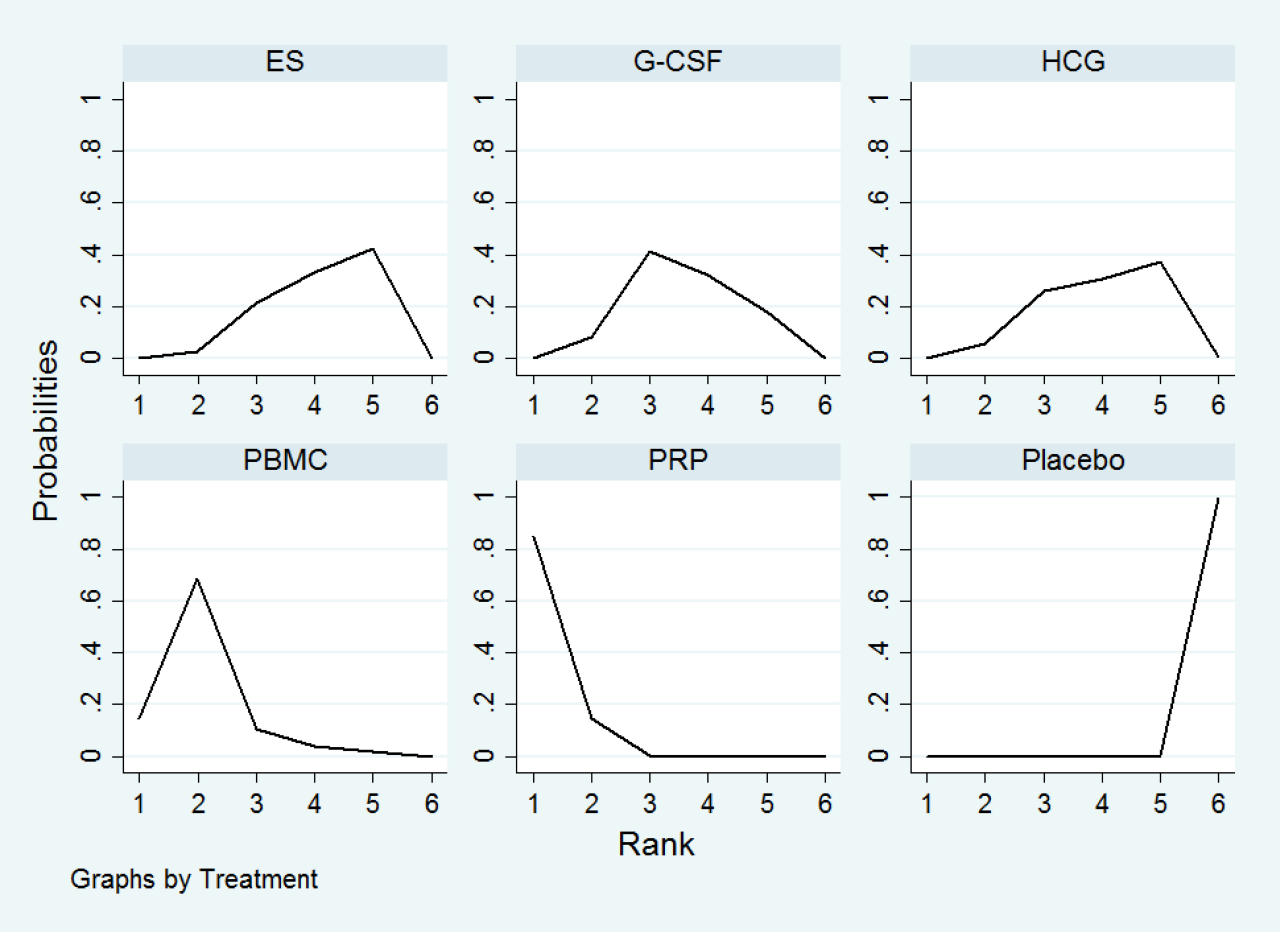
The ranking of intrauterine interventions for clinical pregnancy.

All the 21 RCTs reported clinical pregnancy as an outcome. Overall, 3079 women with two or more implantation failures undergoing fresh or frozen ET were randomized to 6 different treatments including placebo or no treatment. The network plot for clinical pregnancy is shown in **Figure 2**. In addition to control treatment, one RCT(150 women) compared G-CSF with HCG. The remaining comparisons were ES versus control (six RCTs; 1006 women), G-CSF versus control (five RCTs; 547 women), HCG versus control (two RCTs; 255 women), PBMC versus control (three RCTs; 366 women), and PRP versus control (four RCTs; 755 women). The results of the network meta-analysis for clinical pregnancy are shown in **Figure 3**. Network meta-analysis showed that compared with control, PRP, PBMC, G-CSF, HCG, and ES resulted in a higher rate of clinical pregnancy(OR 3.78, 95% CI 2.72 to 5.25; 2.79, 95% CI 1.75 to 4.45; 1.93, 95% CI 1.37 to 2.72; 1.80, 95% CI 1.18 to 2.72; 1.75, 95% CI 1.29 to 2.36, respectively). Inconsistency tests showed no evidence of inconsistency. The SUCRA values for PRP, PBMC, G-CSF, HCG, ES, and placebo were 96.9%, 78.2%, 48.4%, 40.4%, 36.0%, and 0.1%, respectively (**Figure 4**).

#### Secondary outcome: Live birth/ongoing pregnancy

Thirteen studies reported on live birth/ongoing pregnancy rates. The network plot for live birth/ongoing pregnancy is shown in **Supplemental Figure S2**. The most frequent comparisons were ES versus control (five RCTs; 891 women), followed by PRP versus control (three RCTs; 658 women), PBMC versus control (two RCTs; 312 women), G-CSF versus control (two RCTs; 277 women) and HCG versus control (one RCT; 115 women). The network meta-analysis for live birth/ongoing pregnancy is shown in **Supplemental Figure S3**. Network meta-analysis showed that compared with control, PRP, PBMC, and ES resulted in a higher rate of live birth/ongoing pregnancy(OR 5.96, 95% CI 3.38 to 10.52; OR 2.55, 95% CI 1.27 to 5.11; OR 1.70, 95% CI 1.07 to 2.69, respectively). There were no significant differences between any other interventions and placebo treatment. The SUCRA values for PRP, PBMC, G-CSF, HCG, ES, and placebo were 99.3%, 72.1%, 39.2%, 29.0%, 50.4%, and 10.0%, respectively (**Supplemental Figure S4**).

#### Secondary outcome: Miscarriage

Eleven studies reported miscarriage as an outcome. The network plot for miscarriage is shown in **Supplemental Figure S5**. The results of the network meta-analysis for miscarriage are shown in **Supplemental Figure S6**. There were no significant differences between any interventions and placebo treatment. The SUCRA values for PRP, PBMC, G-CSF, HCG, ES, and placebo were 99.3%, 72.1%, 39.2%, 29.0%, 50.4%, and 10.0%, respectively (**Supplemental Figure S7**).

## Discussion

### Summary of findings

RIF imposes a significant psychological and financial burden on infertile couples and represents one of the most challenging tasks in reproductive medicine. Intrauterine interventions seem promising in improving the success rate of women with RIF. However, it is unclear that which is the most effective intrauterine intervention in increasing pregnancy outcomes for these women. In this network meta-analysis, we evaluated various intrauterine interventions for women with two or more previous implantation failures. We included 21 RCTs(3079 women) in this network meta-analysis. We found that compared with placebo, PRP, PBMC, G-CSF, HCG, and ES significantly improved clinical pregnancy. PRP was the most effective intrauterine intervention for clinical pregnancy, followed by PBMC, G-CSF, HCG, and ES. We also found that compared with placebo, PRP, PBMC, and ES significantly increased live birth/ongoing pregnancy. PRP was the most effective intrauterine intervention for live birth/ongoing pregnancy, followed by PBMC, and ES. No significant differences were found for miscarriage.

### Interpretation and implications

In the current network meta-analysis, we found that PRP was the most effective intrauterine intervention to improve clinical pregnancy and live birth/ongoing pregnancy in women with two or more implantation failures. It is well established that PRP is effective and safe in many medical conditions (26), and has been extensively utilized in regenerative medicine for more than 2 decades (27–29). In 2015, Chang et al. reported the first successful application of intrauterine infusion of PRP in reproductive medicine for women with suboptimal endometrium undergoing IVF (30). Although more and more studies have been published with promising results, the exact mechanism of action of PRP in women with recurrent implantation failure still needs to be elucidated. PRP contains numerous molecules such as cytokines, chemokines, cell-adhesion molecules and growth factors (31), which are essential in endometrial receptivity and embryo implantation (32), and dysregulation of these pro-implantation molecules will result in implantation failure (33). It has been hypothesized that intrauterine infusion of PRP might improve endometrial receptivity and promote embryo implantation by modulating the expression of cytokines such as interleukin-1β(IL-1β), IL-6, and IL-8, increasing the expression of estrogen and progesterone receptor, and promoting endometrial cell proliferation (34).

In our study, we found that PBMC was the second best effective intrauterine intervention to improve clinical pregnancy and live birth in women with two or more implantation failures. The idea of using PBMC in women with RIF is based on the rationale that PBMC can regulate the crosstalk between embryo and endometrium and was first brought up by Yoshioka et al. in 2006 (35). However, the exact role of PBMC in the process of embryo implantation is still unclear, and various possible mechanisms have been proposed to explain the effect of PBMC on promoting implantation. PBMC may increase endometrial receptivity and facilitate a more permissive immune environment for implantation by switching uterine immunity from the Th-1 dominant environment to the Th-2 dominant environment (36, 37). PBMC may also promote trophoblast invasion by increasing the production of leukemia inhibitory factor(LIF) and IL-1β (38). Furthermore, PBMC may induce various cytokines, such as IL-1α and tumor necrosis factor -α(TNF-α) to facilitate embryo attachment and invasion (39).

It is well-known that HCG is produced by cytotrophoblast cells to facilitate embryo implantation and support embryo development. We found that intrauterine injection of HCG was also effective in improving clinical pregnancy in women with two or more implantation failures. HCG may enhance endometrial receptivity by stimulating LIF, vascular endothelial growth factor(VEGF), and matrix metalloproteinase-9(MMP-9) while inhibiting insulin-like growth factor binding protein-1(IGFBP-1) (40). HCG may also modulate immune cells, such as natural killer cells, regulatory T cells, Th-1 and Th-2 cells to facilitate trophoblast invasion and maintain maternal-fetal immune tolerance (41–43).

In this network meta-analysis, we demonstrated that intrauterine infusions of G-CSF was effective in improving clinical pregnancy in women with two or more implantation failures. G-CSF may increase phagocytosis and oxidative process, and modulate implantation processes such as endometrial vascular remodeling, local immune environment and cellular adhesion, which is crucial for embryo implantation (44). G-CSF may also regulate macrophages, Th-2 and Treg cells to maintain intrauterine immune tolerance (45, 46).

Since Barash et al. (47) first demonstrated that local endometrial injury in the cycle preceding IVF treatment significantly increased pregnancy rate and more than doubled the live birth rate in 2003, more and more studies have been published. However, the impact of ES on IVF outcome is still subject of debate. In this network meta-analysis, We found that ES increased clinical pregnancy in women with two or more implantation failures, which was in accordance with a previous meta-analysis (48). However, the beneficial effect of ES was not confirmed in women with one previous failed ET (48) or women undergoing a first embryo transfer (49). Therefore, currently, ES may only be used for women with two or more implantation failures in a clinical trial but not in routine clinical practice. Various potential mechanisms have been proposed to explain the role of ES in improving pregnancy outcomes. ES may induce an aseptic inflammation, release cytokines, growth factors, macrophages, and dendritic cells, and delay endometrial maturation to improve endometrial receptivity and promote synchronization between embryo and endometrium (50–52).

### Strengths and limitations

As far as we know, this is the first systematic review and network meta-analysis for overview of all the available evidence comparing various intrauterine interventions for women with two or more implantation failures undergoing ET. We conducted an extensive electronic search for publications without language restrictions. Our network meta-analysis provided a unique opportunity to simultaneously compare the efficacy of various intrauterine interventions by using evidence from indirect comparisons and to rank different treatments in one pooled analysis. Moreover, we reported all the possible major pregnancy outcomes related to ET. Finally, our findings may provide valuable information for future large high quality RCTs to conduct head to head comparisons such as PRP vs PBMC or any other intrauterine interventions for women with RIF. There were also limitations to our study. First, the inclusion criteria and women’s characteristics differed among these studies. The definition of RIF varied in the included studies. The lack of a universally accepted definition of RIF has been an obstacle for studies to investigate various treatments for women with implantation failures. In our study, 9 of the included studies used two or more implantation failures as RIF criteria, while the remaining 12 used three or more implantation failures as RIF criteria. A universally accepted criteria for RIF would make further studies more homogeneous, and more convenient to compare and combine. Therefore, universal RIF criteria should be established as soon as possible. Second, the diversity of treatment protocols including ovarian stimulation, fresh versus frozen ET, and intrauterine interventions would also make studies more heterogeneous. The protocol for intrauterine interventions varied in terms of the dosages of PRP, PBMC, HCG, and G-CSF, the timing of initiation, the number of ES, and the device used for ES. Therefore, a standard procedure should also be established in future studies. Third, many of the studies have had methodological limitations, including small sample size, single center RCTs, and unclear methods of randomization and allocation concealment. Finally, not all the included studies reported live birth, and most of the included studies did not report adverse effects. Despite the evidence, the effectiveness of these intrauterine interventions needs to be confirmed in future large high quality trials, and currently there is no rationale to offer any of the interventions to women in routine clinical practice with the purpose to overcome implantation failure.

## Conclusion

In conclusion, according to current evidence, intrauterine interventions were effective in improving clinical pregnancy in women with two or more implantation failures. PRP was the most effective intrauterine intervention, followed by PBMC, G-CSF, HCG and ES. PRP was also the most effective intrauterine intervention in improving live birth/ongoing pregnancy in these women, followed by PBMC, and ES. These findings indicate that intrauterine interventions may provide an alternative method for women with two or more implantation failures. However, more large high level RCTs are still warranted to confirm our findings and to guide clinical practice.

## Author contributions

XJ conceived the study. YL and DL screened studies, extracted and analyzed the data. XJ performed statistical analysis. XJ wrote the manuscript. All authors contributed to the article and approved the submitted version.

## Conflict of interest

The authors declare that the research was conducted in the absence of any commercial or financial relationships that could be construed as a potential conflict of interest.

## Publisher’s note

All claims expressed in this article are solely those of the authors and do not necessarily represent those of their affiliated organizations, or those of the publisher, the editors and the reviewers. Any product that may be evaluated in this article, or claim that may be made by its manufacturer, is not guaranteed or endorsed by the publisher.

## Supplementary material

Supplementary Figure 1
**(A)** Risk of bias graph **(B)** Risk of bias summary.

Supplemental Figure 2 Network plot for live birth/ongoing pregnancy.

Supplemental Figure 3 The results of the network meta-analysis for live birth/ongoing pregnancy.

Supplemental Figure 4 The ranking of intrauterine interventions for live birth/ongoing pregnancy.

Supplementary Figure 5 Network plot for miscarriage.

Supplementary Figure 6 The results of the network meta-analysis for miscarriage.

Supplementary Figure 7 The ranking of intrauterine interventions for miscarriage.

Supplementary Table 1 Characteristics of the included studies.

## References

[1] Inhorn MC Patrizio P . Infertility around the globe: new thinking on gender, reproductive technologies and global movements in the 21st century. Hum Reprod Update (2015) 21(4):411–26. doi: 10.1093/humupd/dmv016 25801630

[2] Boivin J Bunting L Collins JA Nygren KG . International estimates of infertility prevalence and treatment-seeking: potential need and demand for infertility medical care. Hum Reprod (Oxford England) (2007) 22(6):1506–12. doi: 10.1093/humrep/dem046 17376819

[3] Malizia BA Hacker MR Penzias AS . Cumulative live-birth rates after *in vitro* fertilization. New Engl J Med (2009) 360(3):236–43. doi: 10.1056/NEJMoa0803072 19144939

[4] Toftager M Bogstad J Lossl K Praetorius L Zedeler A Bryndorf T . Cumulative live birth rates after one ART cycle including all subsequent frozen-thaw cycles in 1050 women: secondary outcome of an RCT comparing GnRH-antagonist and GnRH-agonist protocols. Hum Reprod (Oxford England) (2017) 32(3):556–67. doi: 10.1093/humrep/dew358 28130435

[5] Norwitz ER Schust DJ Fisher SJ . Implantation and the survival of early pregnancy. New Engl J Med (2001) 345(19):1400–8. doi: 10.1056/NEJMra000763 11794174

[6] Cimadomo D Craciunas L Vermeulen N Vomstein K Toth B . Definition, diagnostic and therapeutic options in recurrent implantation failure: an international survey of clinicians and embryologists. Hum Reprod (Oxford England) (2021) 36(2):305–17. doi: 10.1093/humrep/deaa317 33313697

[7] Cakiroglu Y Tiras B . Determining diagnostic criteria and cause of recurrent implantation failure. Curr Opin Obstetrics Gynecol (2020) 32(3):198–204. doi: 10.1097/GCO.0000000000000620 32251092

[8] Simon A Laufer N . Repeated implantation failure: clinical approach. Fertil Steril (2012) 97(5):1039–43. doi: 10.1016/j.fertnstert.2012.03.010 22464086

[9] Polanski LT Baumgarten MN Quenby S Brosens J Campbell BK Raine-Fenning NJ . What exactly do we mean by ‘recurrent implantation failure’? a systematic review and opinion. Reprod Biomed Online (2014) 28(4):409–23. doi: 10.1016/j.rbmo.2013.12.006 24581986

[10] Coughlan C Ledger W Wang Q Liu F Demirol A Gurgan T . Recurrent implantation failure: definition and management. Reprod Biomed Online (2014) 28(1):14–38. doi: 10.1016/j.rbmo.2013.08.011 24269084

[11] Zeyneloglu HB Onalan G . Remedies for recurrent implantation failure. Semin Reprod Med (2014) 32(4):297–305. doi: 10.1055/s-0034-1375182 24919029

[12] Margalioth EJ Ben-Chetrit A Gal M Eldar-Geva T . Investigation and treatment of repeated implantation failure following IVF-ET. Hum Reprod (Oxford England) (2006) 21(12):3036–43. doi: 10.1093/humrep/del305 16905766

[13] Cakmak H Taylor HS . Implantation failure: molecular mechanisms and clinical treatment. Hum Reprod Update (2011) 17(2):242–53. doi: 10.1093/humupd/dmq037 PMC3039220 20729534

[14] Evans J Hannan NJ Edgell TA Vollenhoven BJ Lutjen PJ Osianlis T . Fresh versus frozen embryo transfer: backing clinical decisions with scientific and clinical evidence. Hum Reprod Update (2014) 20(6):808–21. doi: 10.1093/humupd/dmu027 24916455

[15] Cavalcante MB Cavalcante C Sarno M Barini R . Intrauterine perfusion immunotherapies in recurrent implantation failures: Systematic review. Am J Reprod Immunol (New York NY: 1989) (2020) 83(6):e13242. doi: 10.1111/aji.13242 32248580

[16] Tapia-Pizarro A Argandona F Palomino WA Devoto L . Human chorionic gonadotropin (hCG) modulation of TIMP1 secretion by human endometrial stromal cells facilitates extravillous trophoblast invasion in vitro. Hum Reprod (Oxford England) (2013) 28(8):2215–27. doi: 10.1093/humrep/det136 23696542

[17] Metcalf D . The granulocyte-macrophage colony-stimulating factors. Sci (New York NY) (1985) 229(4708):16–22. doi: 10.1126/science.2990035 2990035

[18] Demetri GD Griffin JD . Granulocyte colony-stimulating factor and its receptor. Blood (1991) 78(11):2791–808. doi: 10.1182/blood.V78.11.2791.bloodjournal78112791 1720034

[19] Barreda DR Hanington PC Belosevic M . Regulation of myeloid development and function by colony stimulating factors. Dev Comp Immunol (2004) 28(5):509–54. doi: 10.1016/j.dci.2003.09.010 15062647

[20] Xu S Hoglund M Hakansson L Venge P . Granulocyte colony-stimulating factor (G-CSF) induces the production of cytokines *in vivo* . Br J Haematol (2000) 108(4):848–53. doi: 10.1046/j.1365-2141.2000.01943.x 10792294

[21] Daiter E Pampfer S Yeung YG Barad D Stanley ER Pollard JW . Expression of colony-stimulating factor-1 in the human uterus and placenta. J Clin Endocrinol Metab (1992) 74(4):850–8. doi: 10.1210/jcem.74.4.1548350 1548350

[22] Egawa H Fujiwara H Hirano T Nakayama T Higuchi T Tatsumi K . Peripheral blood mononuclear cells in early pregnancy promote invasion of human choriocarcinoma cell line, BeWo cells. Hum Reprod (Oxford England) (2002) 17(2):473–80. doi: 10.1093/humrep/17.2.473 11821298

[23] Marx RE . Platelet-rich plasma (PRP): what is PRP and what is not PRP? Implant dentistry (2001) 10(4):225–8. doi: 10.1097/00008505-200110000-00002 11813662

[24] Hutton B Salanti G Caldwell DM Chaimani A Schmid CH Cameron C . The PRISMA extension statement for reporting of systematic reviews incorporating network meta-analyses of health care interventions: checklist and explanations. Ann Intern Med (2015) 162(11):777–84. doi: 10.7326/M14-2385 26030634

[25] Higgins JP Altman DG Gotzsche PC Juni P Moher D Oxman AD . The cochrane collaboration’s tool for assessing risk of bias in randomised trials. BMJ (Clinical Res ed) (2011) 343:d5928. doi: 10.1136/bmj.d5928 PMC3196245 22008217

[26] Gupta S Paliczak A Delgado D . Evidence-based indications of platelet-rich plasma therapy. Expert Rev Hematol (2021) 14(1):97–108. doi: 10.1080/17474086.2021.1860002 33275468

[27] Marx RE . Platelet-rich plasma: evidence to support its use. J Oral Maxillofac Surgery: Off J Am Assoc Oral Maxillofac Surg (2004) 62(4):489–96. doi: 10.1016/j.joms.2003.12.003 15085519

[28] Foster TE Puskas BL Mandelbaum BR Gerhardt MB Rodeo SA . Platelet-rich plasma: from basic science to clinical applications. Am J Sports Med (2009) 37(11):2259–72. doi: 10.1177/0363546509349921 19875361

[29] Marx RE Carlson ER Eichstaedt RM Schimmele SR Strauss JE Georgeff KR . Platelet-rich plasma: Growth factor enhancement for bone grafts. Oral Surg Oral Med Oral Pathol Oral Radiol Endod (1998) 85(6):638–46. doi: 10.1016/S1079-2104(98)90029-4 9638695

[30] Chang Y Li J Chen Y Wei L Yang X Shi Y . Autologous platelet-rich plasma promotes endometrial growth and improves pregnancy outcome during *in vitro* fertilization. Int J Clin Exp Med (2015) 8(1):1286–90. PMC4358582 25785127

[31] Wu PI Diaz R Borg-Stein J . Platelet-rich plasma. Phys Med Rehabil Clinics North America (2016) 27(4):825–53. doi: 10.1016/j.pmr.2016.06.002 27788903

[32] Lessey BA . The role of the endometrium during embryo implantation. Hum Reprod (Oxford England) (2000) 15 Suppl 6:39–50. 11261482

[33] Dimitriadis E White CA Jones RL Salamonsen LA . Cytokines, chemokines and growth factors in endometrium related to implantation. Hum Reprod Update (2005) 11(6):613–30. doi: 10.1093/humupd/dmi023 16006437

[34] Mouanness M Ali-Bynom S Jackman J Seckin S Merhi Z . Use of intra-uterine injection of platelet-rich plasma (PRP) for endometrial receptivity and thickness: a literature review of the mechanisms of action. Reprod Sci (2021) 28(6):1659–70. doi: 10.1007/s43032-021-00579-2 33886116

[35] Yoshioka S Fujiwara H Nakayama T Kosaka K Mori T Fujii S . Intrauterine administration of autologous peripheral blood mononuclear cells promotes implantation rates in patients with repeated failure of IVF-embryo transfer. Hum Reprod (Oxford England) (2006) 21(12):3290–4. doi: 10.1093/humrep/del312 17021188

[36] Hashii K Fujiwara H Yoshioka S Kataoka N Yamada S Hirano T . Peripheral blood mononuclear cells stimulate progesterone production by luteal cells derived from pregnant and non-pregnant women: possible involvement of interleukin-4 and interleukin-10 in corpus luteum function and differentiation. Hum Reprod (Oxford England) (1998) 13(1O):2738–44. doi: 10.1093/humrep/13.10.2738 9804222

[37] Ginsburg ES Xiao L Gargiulo AR Kung FT Politch JA Schust DJ . T-Helper 2 and 3 type immunity to trophoblast in successful *in vitro* fertilization-embryo transfer. Fertil Steril (2005) 83(6):1659–64. doi: 10.1016/j.fertnstert.2004.12.038 15950633

[38] Yu N Yan W Yin T Wang Y Guo Y Zhou D . HCG-activated human peripheral blood mononuclear cells (PBMC) promote trophoblast cell invasion. PloS One (2015) 10(6):e0125589. doi: 10.1371/journal.pone.0125589 26087261 PMC4472760

[39] Fujiwara H . Do circulating blood cells contribute to maternal tissue remodeling and embryo-maternal cross-talk around the implantation period? Mol Hum Reprod (2009) 15(6):335–43. doi: 10.1093/molehr/gap027 19346239

[40] Licht P Fluhr H Neuwinger J Wallwiener D Wildt L . Is human chorionic gonadotropin directly involved in the regulation of human implantation? molecular and cellular endocrinology. Mol Cell Endocrinol (2007) 269(1-2):85–92. doi: 10.1016/j.mce.2006.09.016 17367920

[41] Schumacher A Brachwitz N Sohr S Engeland K Langwisch S Dolaptchieva M . Human chorionic gonadotropin attracts regulatory T cells into the fetal-maternal interface during early human pregnancy. J Immunol (Baltimore Md: 1950) (2009) 182(9):5488–97. doi: 10.4049/jimmunol.0803177 19380797

[42] Filicori M Fazleabas AT Huhtaniemi I Licht P Rao Ch V Tesarik J . Novel concepts of human chorionic gonadotropin: reproductive system interactions and potential in the management of infertility. Fertil Steril (2005) 84(2):275–84. doi: 10.1016/j.fertnstert.2005.02.033 16084861

[43] Diao LH Li GG Zhu YC Tu WW Huang CY Lian RC . Human chorionic gonadotropin potentially affects pregnancy outcome in women with recurrent implantation failure by regulating the homing preference of regulatory T cells. Am J Reprod Immunol (New York NY: 1989) (2017) 77(3). doi: 10.1111/aji.12618 28044377

[44] Rahmati M Petitbarat M Dubanchet S Bensussan A Chaouat G Ledee N . Granulocyte-colony stimulating factor related pathways tested on an endometrial *ex-vivo* model. PLoS One (2014) 9(9):e102286. doi: 10.1371/journal.pone.0102286 25275446 PMC4183482

[45] Moldenhauer LM Keenihan SN Hayball JD Robertson SA . GM-CSF is an essential regulator of T cell activation competence in uterine dendritic cells during early pregnancy in mice. J Immunol (Baltimore Md: 1950) (2010) 185(11):7085–96. doi: 10.4049/jimmunol.1001374 20974989

[46] Rutella S Zavala F Danese S Kared H Leone G . Granulocyte colony-stimulating factor: a novel mediator of T cell tolerance. J Immunol (Baltimore Md: 1950) (2005) 175(11):7085–91. doi: 10.4049/jimmunol.175.11.7085 16301609

[47] Barash A Dekel N Fieldust S Segal I Schechtman E Granot I . Local injury to the endometrium doubles the incidence of successful pregnancies in patients undergoing *in vitro* fertilization. Fertil Steril (2003) 79(6):1317–22. doi: 10.1016/s0015-0282(03)00345-5 12798877

[48] Vitagliano A Di Spiezio Sardo A Saccone G Valenti G Sapia F Kamath MS . Endometrial scratch injury for women with one or more previous failed embryo transfers: a systematic review and meta-analysis of randomized controlled trials. Fertil Steril (2018) 110(4):687–702.e2. doi: 10.1016/j.fertnstert.2018.04.040 30196966

[49] Vitagliano A Andrisani A Alviggi C Vitale SG Valenti G Sapia F . Endometrial scratching for infertile women undergoing a first embryo transfer: a systematic review and meta-analysis of published and unpublished data from randomized controlled trials. Fertil Steril (2019) 111(4):734–46.e2. doi: 10.1016/j.fertnstert.2018.12.008 30683590

[50] Granot I Gnainsky Y Dekel N . Endometrial inflammation and effect on implantation improvement and pregnancy outcome. Reprod (Cambridge England) (2012) 144(6):661–8. doi: 10.1530/REP-12-0217 23028125

[51] Gnainsky Y Granot I Aldo PB Barash A Or Y Schechtman E . Local injury of the endometrium induces an inflammatory response that promotes successful implantation. Fertil Steril (2010) 94(6):2030–6. doi: 10.1016/j.fertnstert.2010.02.022 PMC3025806 20338560

[52] Kalma Y Granot I Gnainsky Y Or Y Czernobilsky B Dekel N . Endometrial biopsy-induced gene modulation: first evidence for the expression of bladder-transmembranal uroplakin ib in human endometrium. Fertil Steril (2009) 91(4):1042–9, 9.e1-9. doi: 10.1016/j.fertnstert.2008.01.043 18355812

