# Supplementary figures and images for: Intrauterine interventions for women with two or more implantation failures: A systematic review and network meta-analysis
Source: Front Endocrinol (Lausanne). 2022 Aug 29;13:959121. doi: 10.3389/fendo.2022.959121 (PMC9464901; doi:10.3389/fendo.2022.959121)

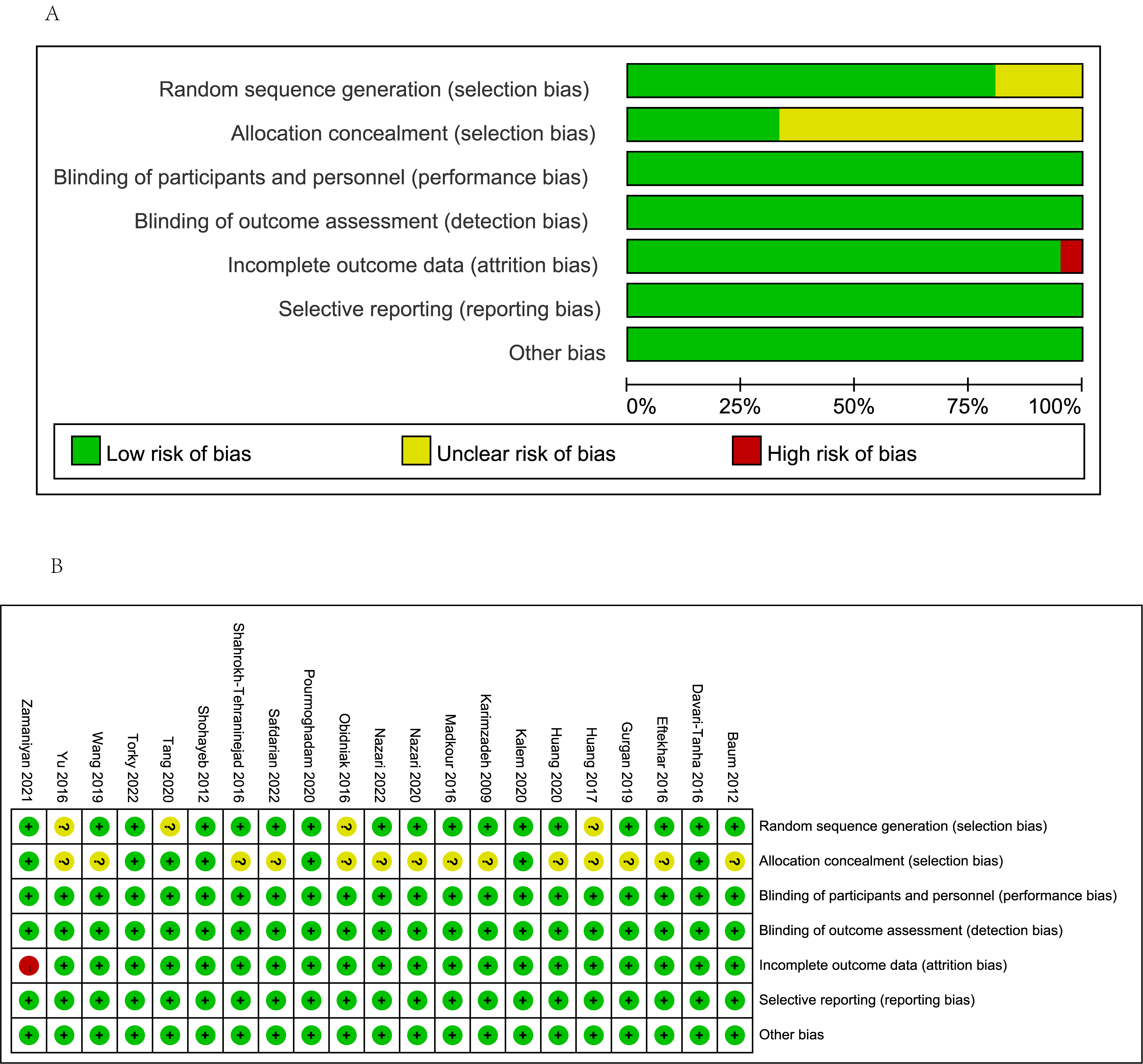

Supplement: Supplementary Figure 1 — (A) Risk of bias graph (B) Risk of bias summary. [file Image_1.tif]

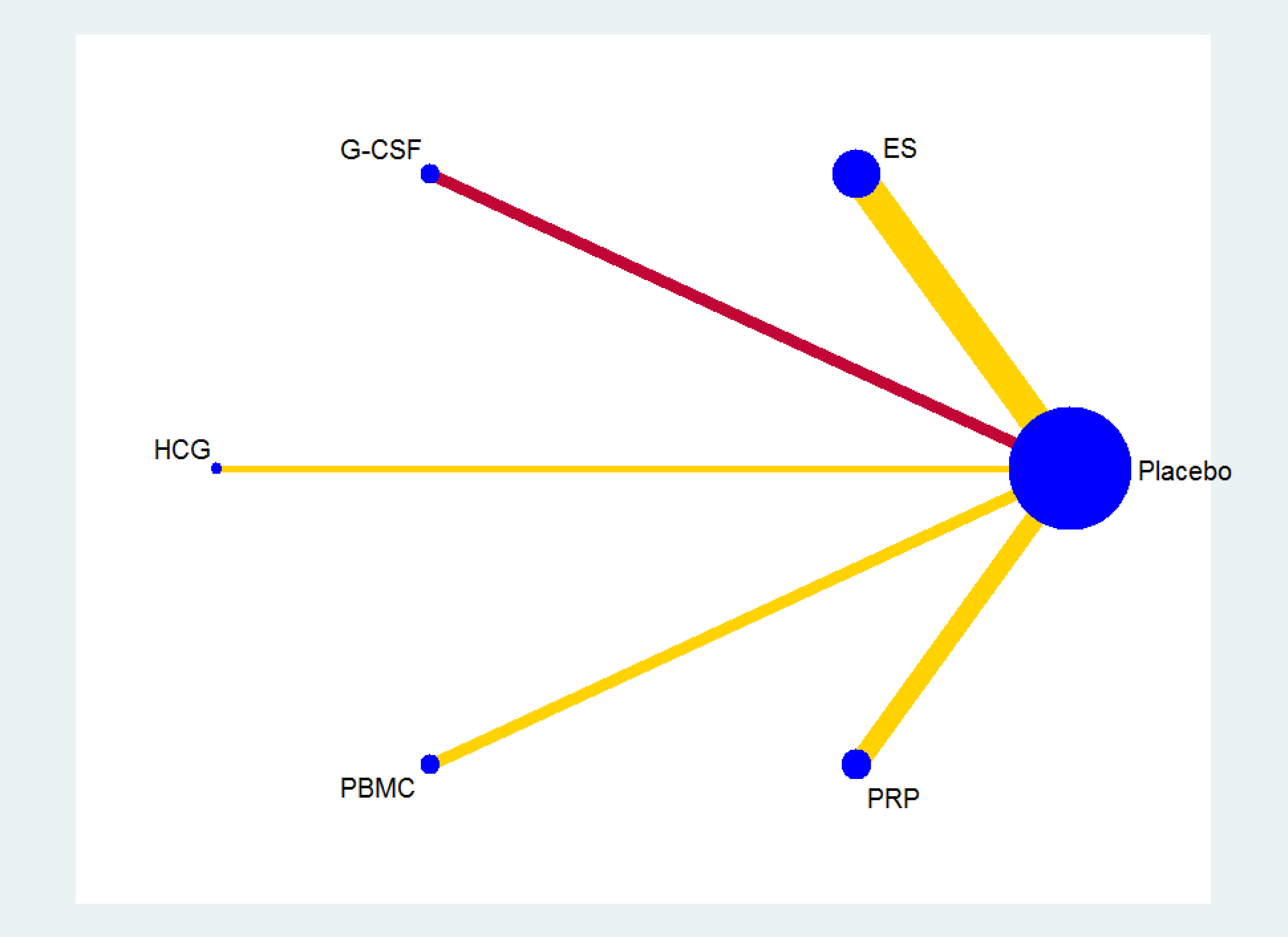

Supplement: Supplemental Figure 2 — Network plot for live birth/ongoing pregnancy. [file Image_2.tif]

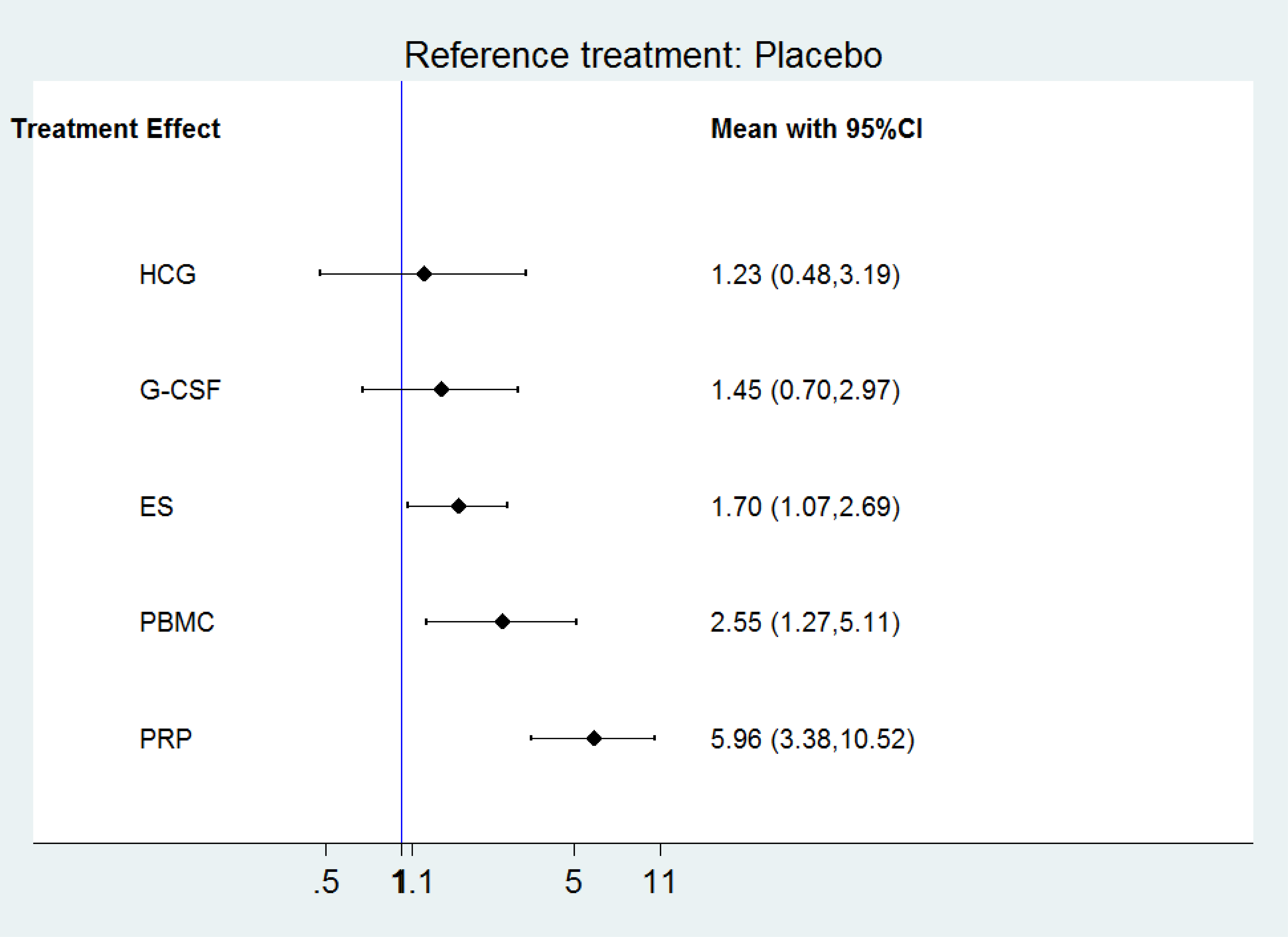

Supplement: Supplemental Figure 3 — The results of the network meta-analysis for live birth/ongoing pregnancy. [file Image_3.tif]

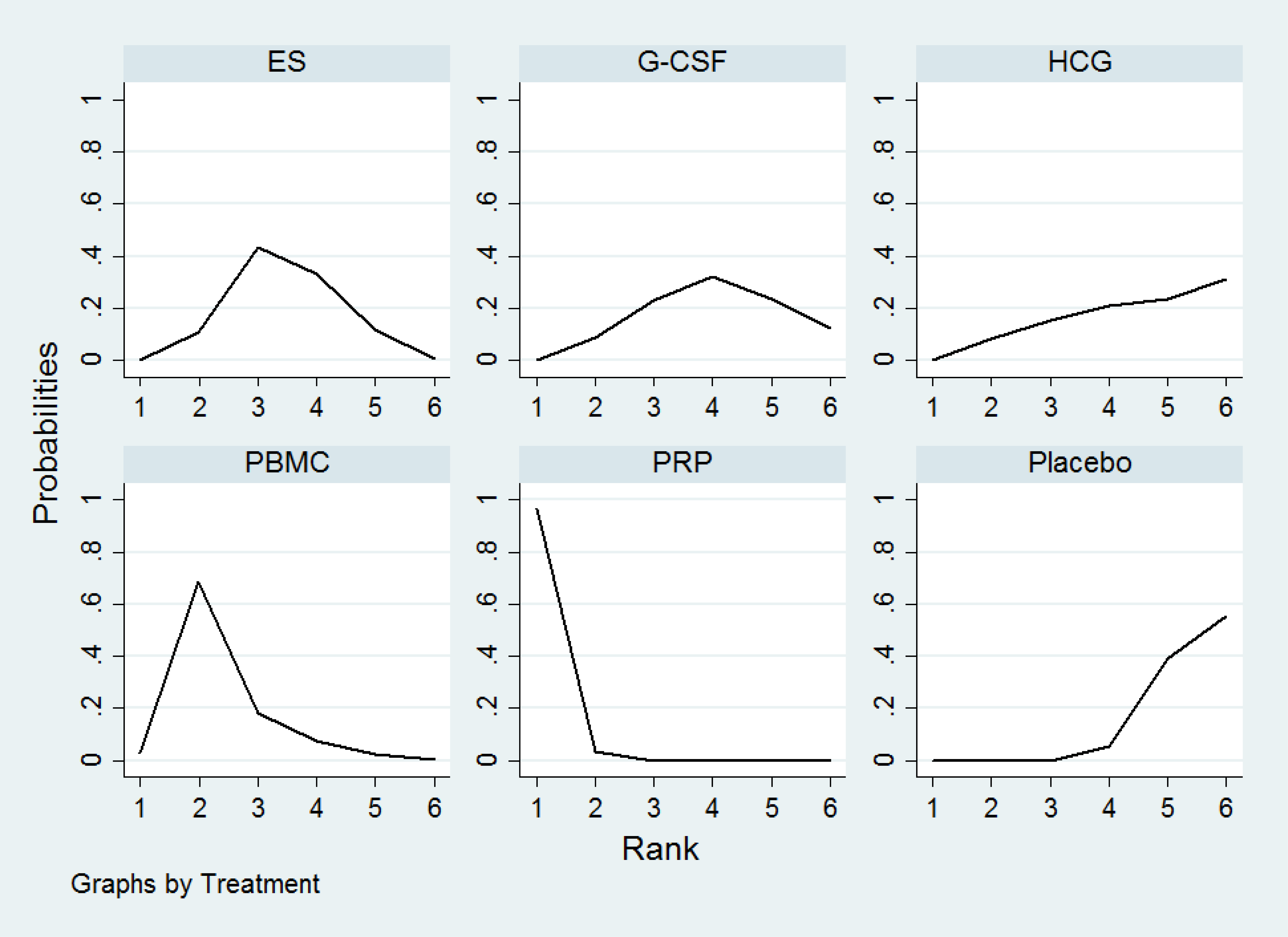

Supplement: Supplemental Figure 4 — The ranking of intrauterine interventions for live birth/ongoing pregnancy. [file Image_4.tif]

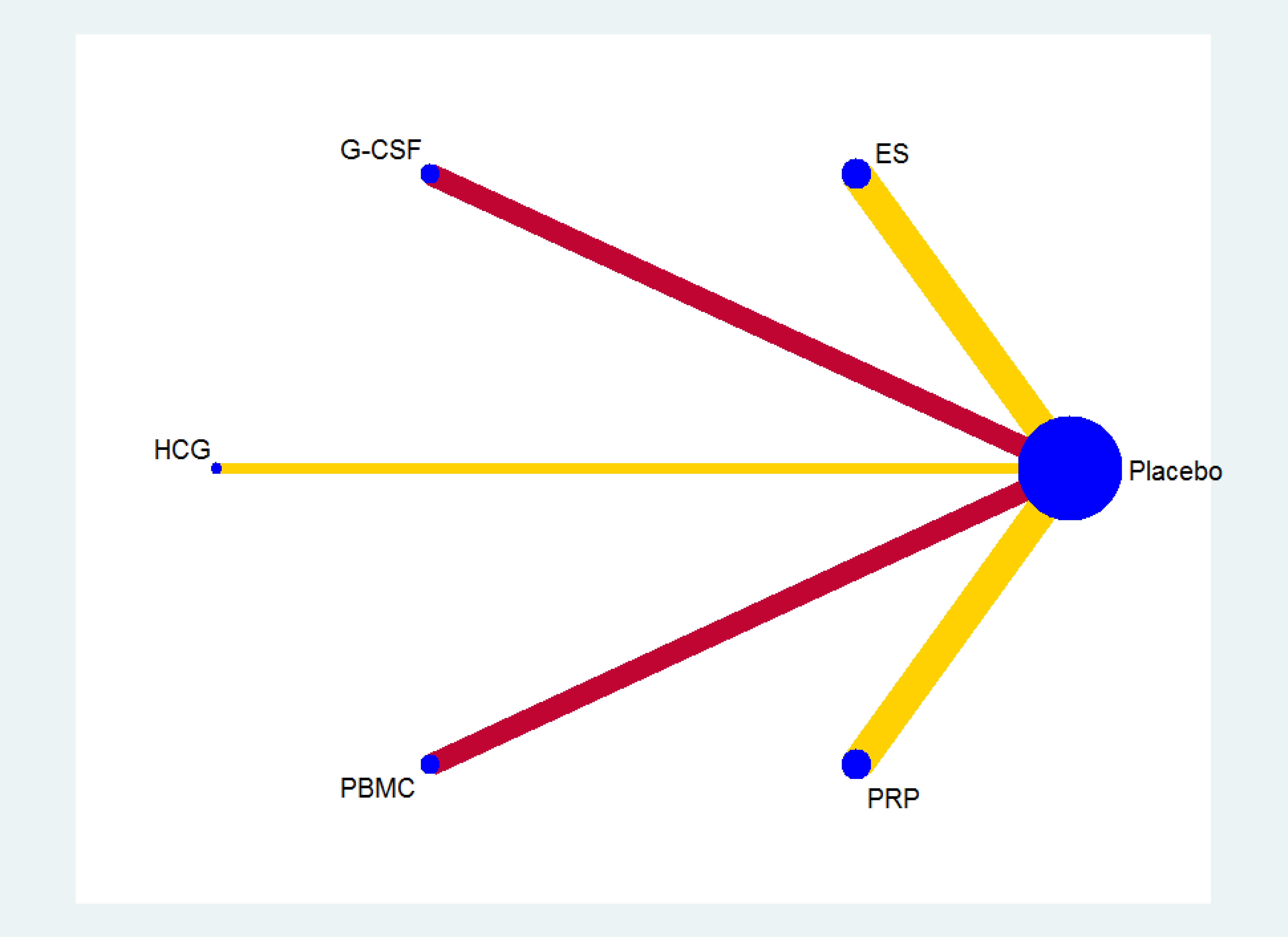

Supplement: Supplementary Figure 5 — Network plot for miscarriage. [file Image_5.tif]

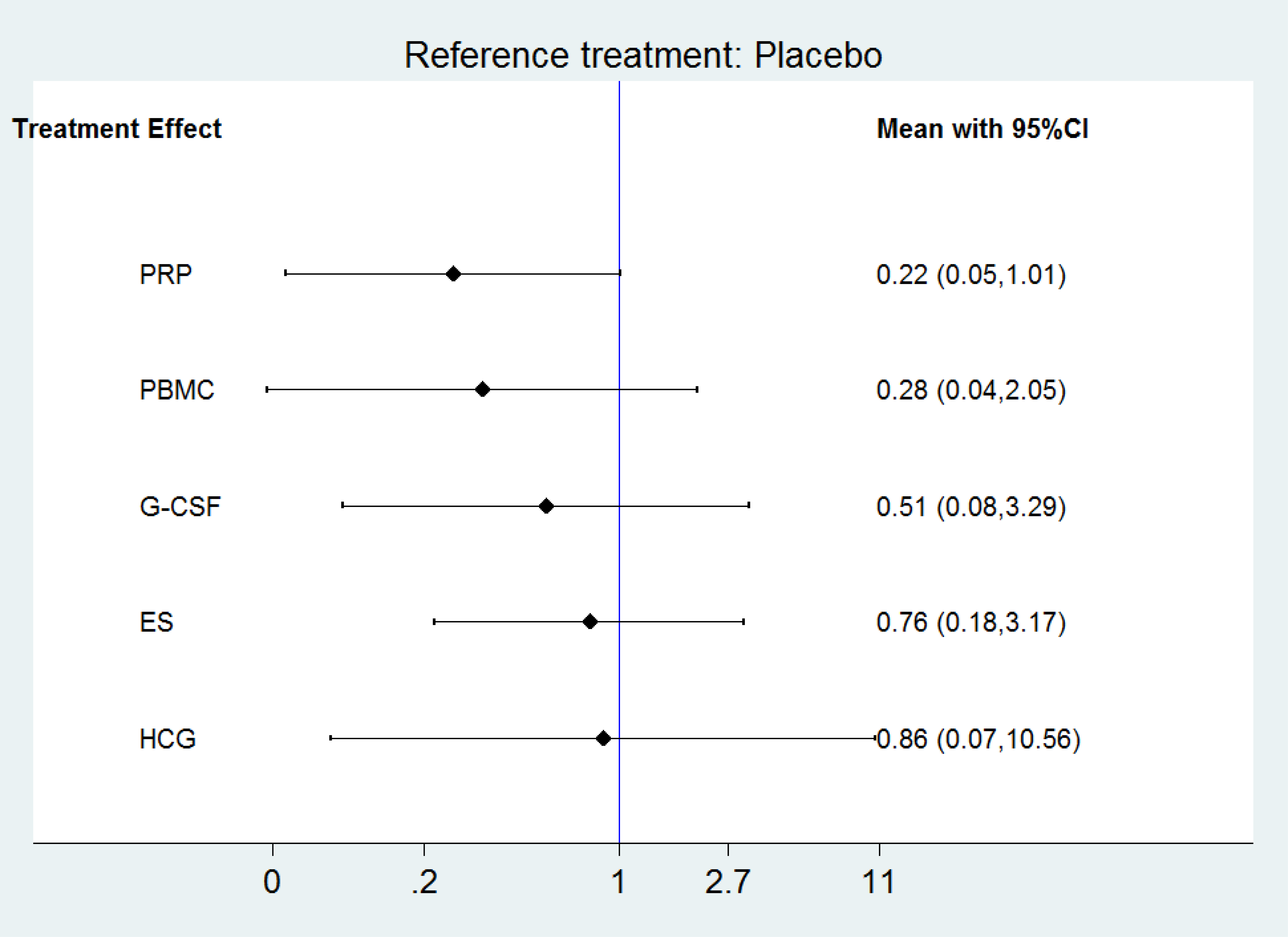

Supplement: Supplementary Figure 6 — The results of the network meta-analysis for miscarriage. [file Image_6.tif]

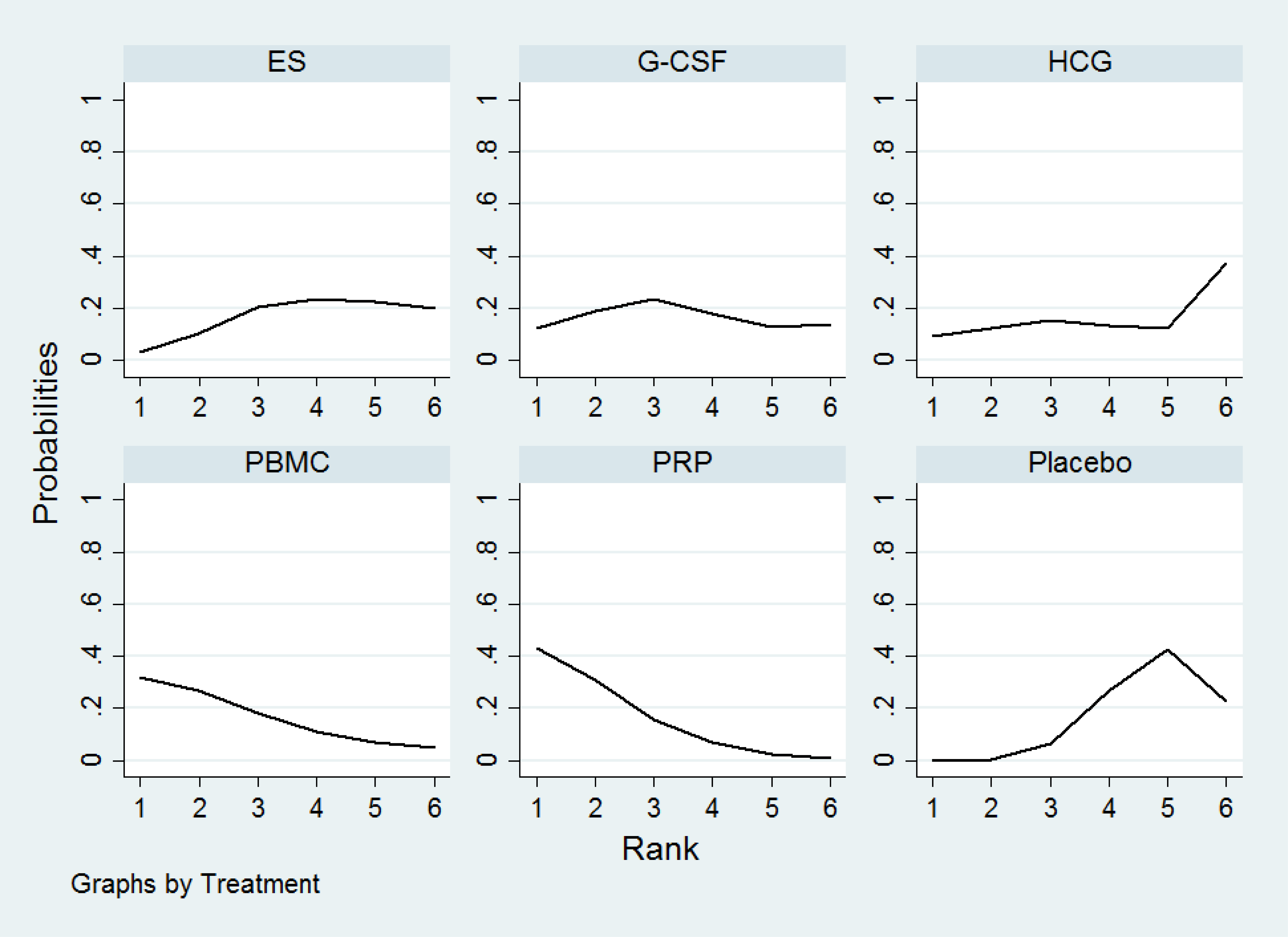

Supplement: Supplementary Figure 7 — The ranking of intrauterine interventions for miscarriage. [file Image_7.tif]
