# Supplementary material for: Intrauterine interventions for women with two or more implantation failures: A systematic review and network meta-analysis
Source: Front Endocrinol (Lausanne). 2022 Aug 29;13:959121. doi: 10.3389/fendo.2022.959121 (PMC9464901; doi:10.3389/fendo.2022.959121)
Supplement: Supplementary Table 1 — Characteristics of the included studies. [file Table_1.docx]

　　 Supplemental Table 1. Characteristics of the included studies.

| **Trial(year)** | **Region** | **Study design** | **Inclusion criteria** | **Exclusion criteria** | **Age(Intevention vs control)** | **Number of previous implantation failure(Intervention vs control)** | **Intervention** | **Control** | **Ovarian stimulation** | **IVF/ICSI treatment** | **Pregnancy outcomes** |
| --- | --- | --- | --- | --- | --- | --- | --- | --- | --- | --- | --- |
| **Baum et al. 2012(1)** | Israel | Single center RCT | Women between 18-41 years with RIF undergoing IVF with fresh embryo transfer, RIF defined as three or more previous IVF-ET failures with good ovarian response. | Uterine malformation, endometrioma, and hydrosalpinx. | 34.8±4.3 vs 34.4±5.4 | 8.5±3.5 vs 8.8±4.6 | Endometrial biopsy on days 9-12 and 21-24 of the menstrual cycle preceding the IVF treatment cycle | Placebo(cervical pipelle without scraping or biopsy) | Long agonist, antagonist protocol or short agonist protocol | Fresh IVF-ET | Clinical pregnancy rate, live birth rate |
| **Gurgan et al. 2019(2)** | Turkey | Single center RCT | Women with RIF and FSH ≤ 15 IU/mL undergoing ICSI, RIF defined as the failure of achieving a clinical pregnancy after the transfer of at least four good-quality embryos in a minimum of three fresh or frozen cycles to a woman under the age of 40 years. | Congenital uterine anomalies, Asherman's syndrome, uterine cavity distorted by myoma or endometrial polyps, endometriosis, endometrioma, endometrial thickness ＜7mm, BMI of ＜18.5 or ＞29.9. | 34.31±3.83 vs 33.64±4.25 | 3.79±2.09 vs 3.18±1.55 | Endometrial injury on days 10-12 of the menstrual cycle preceding the ICSI treatment cycle. | No intervention | Long agonist, or antagonist protocol | Fresh /frozen ICSI | Clinical pregnnacy rate, live birth rate, HCG positivity rate |
| **Karimzadeh et al. 2009(3)** | Iran | Single center RCT | Women between 20-40 years with RIF and without history of blood diseases undergoing IVF/ICSI, RIF defined as 2-6 IVF-ET cycles and the transfer of at least 10 high-grade embryos per woman without clinical pregnancy. | Women ＞ 40 years, poor responders in previous cycles, uterine malformation, endometrioma, hydrosalpinx. | 29.96±3.93 vs 29.73±3.92 | 2.52±1.42 vs 2.18±0.54 | Endometrial biopsy on days 21-26 of the menstrual cycle preceding the IVF/ICSI treatment cycle. | No intervention | Long agonist protocol | Fresh IVF/ICSI | Clinical pregnancy rate |
| **Shahrokh-Tehraninejad**  **et al. 2016(4)** | Iran | Single center RCT | Women ＜40 years undergoing frozen IVF , previous at least two IVF/ICSI failures, presence of at least four good-quality embryos, and normal uterus with ＞7mm endometrial thickness. | Myoma ＞5cm, endometrioma ≥3cm, hydrosalpinx, endometrial tuberculosis, previous history of tuberculosis treatment, Asherman's syndrome, BMI ＞30, active vaginal or cervical infection, diabetes, systemic lupus erythematous. | 29.5±6.4 vs 28.3±5.6 | 2.3±0.5 vs 2.8±0.7 | Endoemtrial crashing on the day 21 of the cycle before IVF cycle | No intervention | GnRH antagonist protocol | Frozen IVF-ET | Clinical pregnancy rate, live birth rate, miscarriage rate, ectopic pregnancy rate. |
| **Shohayeb and EI-Khayat 2012(5)** | Egypt, Saudi Arabia | Double center RCT | Women ＜39 years with RIF undergoing ICSI, RIF defined as the failure to achieve pregnancy after2-6 ICSI cycles with the transfer of more than 10 high-grade embryos. | Submucous myoma, endometrial polyp, Asherman's syndrome, septate or bicornuate uterus. | 30.7±4.5 vs 30.6±4.5 | Not stated | Endometrial scraping on days 4-7 of the cycle before ICSI cycle | Hysteroscopy without endometrial scraping | Long, short , or antagonist protocol | Fresh ICSI | Clinical pregnancy rate, live birth, miscarriage rate. |
| **Tang et al. 2020(6)** | China | Single center RCT | Women with basal serum progesterone＜1.2 ng/mL, ≥2 previous implantation failures, and normal uterine cavity undergoing frozen-thawed ET. | Pelvic surgery history, difficult ET history, ＞40 years, BMI ＞27, hydrosalpinx, endometriosis, severe intrauterine adhesion, uterine polyp, submucous myoma and recent oral contraception use. | 30.98±3.65 vs 30.84±3.95 | 2.57±1.15 vs 2.55±1.07 | Endoemtrial scratching on the day 3 of the cycle before ET cycle | No intervention | Not stated | Frozen ET | Clinical pregnancy rate, live birth rate, biochemical pregnancy rate, multiple pregnancy rate, miscarriage rate, ectopic pregnancy rate. |
| **Davari-Tanha et al. 2016(7)** | Iran | Double center RCT | Women ＜40 years with RIF undergoing fresh or frozen embryo transfer, RIF defined as 3 previous implantation failures with the transfer of ≥ 4 good-quality embryos without uterine or thrombophilic factors | Women with history of renal disease, sickle cell disease, malignancy, or sensitivity of G-CSF | 35.5±4.32 vs 35.3±3.98 | 3.5±2.1 vs 4.2±1.5 | One ml(300 μg) G-CSF was injected into uterine cavity on the day of oocyte retrieval in fresh embryo transfer cycle, or on the day of starting progesterone in frozen embryo transfer cycle. | One ml normal saline was injected into uterine cavity on the day of oocyte retrieval in fresh embryo transfer cycle, or on the day of starting progesterone in frozen embryo transfer cycle  Placebo group(a catheter through cervix without any injection) | Not stated | Fresh /frozen ET | Clinical pregnancy rate, miscarriage rate, chemical pregnancy rate. |
| **Eftekhar et al. 2016(8)** | Iran | Single center RCT | Women between 20-40 years with RIF undergoing embryo transfer, RIF defined as defined as after 2-6 transfers of ≥ 10 high-grade embryos without pregnancy | Women with sickle cell disease, chronic neutropenia, malignancy history, renal failure, congenital fructose intolerance, respiratory infection, endometriosis, or severe male factor, | 32.55±4.61 vs 31.75±5.16 | 2.57±1.69 vs 3.41±1.54 | 0.5 ml(300 μg) G-CSF was injected into uterine cavity on the day of oocyte retrieval | No intervention | Not stated | Fresh  ET | Clinical pregnancy rate |
| **Huang et al. 2020(9)** | China | Single center RCT | Women with ≥ 2 implantation failures with the transfer of ≥ 1 high-quality embryo each time, primary infertility, ≤38 years, BMI between 18-24, normal enmetrial thickness(8-16mm), and ≥ 2 frozen embryos available(including ≥ 1 good-quality embryo or good-quality blastocyst) | Endometrial polyp, intrauterine adhesion, uterine submucosal myoma, chronic endometritis, adenomyosis, hydrosalpinx, polycystic ovary syndrome, ≥ stage 3 endometriosis. | 32.09±4.21 vs 32.07±4.36 | 2.92±0.96 vs 2.94±0.79 | One ml(150 μg) G-CSF was injected into uterine cavity 3 days before embryo transfer | Normal saline was injected into uterine cavity 3 days before embryo transfer | Not stated | Frozen ET | Clinical pregnancy rate, live birth rate, miscarriage rate. |
| **Kalem et al. 2020(10)** | Turkey | Single center RCT | Women with RIF and FSH ≤ 15 IU/mL undergoing ICSI, RIF defined as the failure of achieving a clinical pregnancy after the transfer of at least four good-quality embryos in a minimum of three fresh or frozen cycles to a woman under the age of 40 years. | Congenital uterine anomalies, autoimune diseases, Asherman's syndrome, uterine cavity distorted by myoma or endometrial polyps, endometrial thickness ＜7mm endometriosis, endometrioma, active infection, kidney disease, sickle cell anemia, malignancies, chronic neutropenia. | 34.61±4.77 vs 34.92±5.60 | 3.08±0.27 vs 3.03±0.16 | One ml(30 mIU) G-CSF was infused into uterine cavity on HCG day | One ml normal saline was infused into uterine cavity on HCG day | Long agonist, or antagonist protocol | Fresh ICSI | Clinical pregnnacy rate, live birth rate, miscarriage rate, chemical pregnancy rate, HCG positivity rate |
| **Obidniak**  **et al. 2016(11)** | Russia | RCT | Women between 32-40 years with RIF undergoing frozen-thawed embryo transfer, RIF defined as ≥ 2 IVF cycle with good-quality embryos transferred in each cycle without achieving a clinical pregnancy. | Congenital uterine anomalies, Asherman's syndrome, endometrial thickness ＜7mm | Not stated | Not stated | One ml(30 mIU) G-CSF was infused into uterine cavity 5 days before embryo transfer  or  One ml(30 mIU) G-CSF was injected subcutaneously at the day of ET | No intervention | Not stated | Frozen ET | Clinical pregnancy rate |
| **Torky et al. 2022(12)** | Egypt | Multicenter  RCT | Women between 20 and 39 years undergoing IVF/ICSI with RIF(≥ 3 failures with ≥ 4 good embryos transferred) | Hypersensitivity, sickle cell nephropathy, malignancy history, poor quality embryos, and at risk of ovarian hyperstimulation syndrome. | 35.1±5.04(G-CSF) vs 35.33±5.11(HCG) vs 35.17±4.23(Placebo) | 3.7±1.09 (G-CSF) vs 4.43±0.94(HCG) vs 3.67±0.81(Placebo) | One ml(100μg ) G-CSF or 1ml(500IU)HCG was infused into uterine after oocyte retrieval. | One ml normal saline solution was infused into uterine after oocyte retrieval | Long agonist protocol | Fresh IVF/ICSI | Clinical pregnancy rate, chemical pregnancy rate,  miscarriage rate. |
| **Huang et al. 2016(13)** | China | Single center RCT | Women with ≥ 2 transfer failures with good-quality embryos, ≤38 years, BMI between 18-24, normal enmetrial thickness(8-16mm), undergoing frozen-thawed embryo transfer, and ≥ 2 frozen embryos available(including ≥ 1 good-quality embryo) | Endometrial polyp, intrauterine adhesion, uterine submucosal myoma, adenomyosis, hydrosalpinx, polycystic ovarian syndrome, ≥ stage 3 endometriosis. | 33.95±4.14 vs 33.08±4.38 | 2.40±0.94 vs 2.32±0.75 | One ml(1000 mIU) hCG was infused into uterine cavity 3 days before embryo transfer | One ml normal saline was infused into uterine cavity 3 days before embryo transfer | Not stated | Frozen ET | Clinical pregnancy rate, ongoing pregnancy rate, miscarriage rate. |
| **Wang et al. 2019(14)** | China | Single center RCT | Women ＜40 years with RIF undergoing frozen-thawed embryo transfer, regular menstrual cycles, RIF defined as the failure of achieving a clinical pregnancy after the transfer of at least four good-quality embryos in a minimum of three fresh or frozen cycles | Intramural myoma ≥3cm, submucosal myoma, endometrial polyp, adenomyosis , hydrosalpinx, Asherman's syndrome, endometrial hyperplasia endometriosis, chromosomal abnormalities in either or both of the couple, blastocyst transfer**,** embryo transfer with pre-implantation genetic diagnosis. | 31.35±3.18 vs 31.70±3.56 | 4.14±0.39 vs 4.18±0.42 | 40 μL culture medium with 500 IU hCG was infused into uterine cavity on the day of embryo transfer | 40 μL culture medium was infused into uterine cavity on the day of embryo transfer | Not stated | Fresh  ET | Clinical pregnancy rate, ectopic pregnancy rate. |
| **Madkour et al. 2015(15)** | Morocco | Single center RCT | Women＜40 years undergoing IVF/ICSI with ≥ 2 previous implantation failures after IVF/ICSI, primary infertility, endometrial thickness＜6mm, regular menstrual cycles, BMI ＜30, without infectious negative balance. | Uterine pathology and PCOS. | 34.74±4.17 vs 34.44±3.86 | 3.19±1.75 vs 3.63±1.76 | 1×10^6^ PBMC in 0.4 ml were transferred into uterine cavity 2 days before embryo transfer | No intervention | Antagonist protocol | Fresh IVF/ICSI | Clinical pregnancy rate, miscarriage rate |
| **Pourmoghadam et al. 2020(16)** | Iran | Single center RCT | Women ＜45 years with ≥ 3 previous IVF-ET failures, primary infertility, regular menstrual cycles, and BMI ＜30, RIF defined as a frequent transmission of morphologically good-quality embryos into normal uterus without attaining clinical pregnancy. | Ovulatory disorders, uterine pathology and anomalies, tubal factors, poor ovarian reserve, chromosomal abnormalities, auto-antibodies presence. | 33.42±3.1 vs 34.64±3.0 | 4.5±2.25 vs 4.2±2.33 | 1.5-2×10^7^ PBMC in 500 μL PBS were transferred into uterine cavity 2 days before embryo transfer | 500 μL PBS were transferred into uterine cavity 2 days before embryo transfer | Antagonist protocol | Frozen ET | Clinical pregnancy rate, live birth rate, miscarriage rate. |
| **Yu et al.**  **2016(17)** | China | Single center RCT | Women ＜35 years with ≥ 3 previous IVF-ET failures undergoing frozen-thawed embryo transfer, FSH ≤ 15 IU/mL, | Hydrosalpinx, chromosomal abnormalities, Asherman's syndrome, dysontogenesis, endometrial organic diseasse, and severe endometriosis. | 31.08±3.95 vs 31.22±5.12 | 3.36±1.28 vs 3.44±2.12 | 1-2×10^7^ PBMC in 200 μL RPMI 1640 were transferred into uterine cavity 2 days before embryo transfer | No intervention | Long or short protocol | Frozen ET | Clinical pregnancy rate, live birth rate, miscarriage rate. |
| **Nazari et al. 2020(18)** | Iran | Single center RCT | Women ＜40 years, failed to conceive after ≥ 3 previous embryo transfer with high-quality embryos, undergoing frozen-thawed embryo transfer, BMI＜30. | Uterine abnormalities, hormonal disorders, immunological and hematological disorders, azoospermia, testicular sperm extraction or aspiration, anatomical disorders of the male genital tract, varicocele, and chromosomal abnormalities in the couples. | 35.73±3.49 vs 34.95±4.23 | 5.38±2.3 vs 4.97±2.8 | Intrauterine infusion of 0.5 ml PRP 48h before embryo transfer | No intervention | Not stated | Frozen ET | Clinical pregnancy rate, chemical pregnancy rate |
| **Nazari et al. 2022(19)** | Iran | Single center RCT | Women between 18 and 38, ≥ 3 previous embryo transfer failures with high-quality embryos body mass index ≤30kg/m^2^ ,FSH ≤ 15 IU/mL. | Immunological abnormality, inflammatory conditions, hormonal or anatomical disorders, PCOS, OHSS, history of miscarriage or ectopic pregnancy, myomas, polyps, adhesions, pelvic surgeries, failured fertilization, ＜2 embryos for transfer | 34.11±3.75 vs 33.61±4.06 | 4.1±0.3 vs 4.2±0.5 | Intrauterine infusion of 0.5 ml PRP 48h before blastocyst transfer. | No intervention | Antagonist protocol | Frozen ICSI | Clinical pregnancy rate, chemical pregnancy rate, multiple pregnancy rate, live birth rate. |
| Safdarian  et al. 2022(20) | Iran | Single center RCT | Women between 20 and 40 with ≥ 3 previous embryo transfer with high-quality embryos and ≥ 1 frozen good quality blastocyst embryo undergoing FET. | Chromosomal and genetic disorders, hematological and immunological disorders, hormonal disorders, uterine abnormality , BMI ≥30 kg/ m2, severe endometriosis, and cancellation of ET due to a thin endometrium (≤7 mm). | 33.4±4.9 vs 34±3.73 | Not stated | Intrauterine infusion of 0.5 ml PRP 48h before embryo transfer | No intervention | Not stated | Frozen ET | Clinical pregnancy rate, chemical pregnancy rate, multiple pregnancy rate,ongoing pregnancy, miscarriage. |
| **Zamaniyan**  **et al. 2021(21)** | Iran | Single center RCT | Women between 20-40 years, failed to conceive after ≥ 3 previous embryo transfer with high-quality embryos, undergoing frozen-thawed embryo transfer, BMI＜30, and normal hysterosalpingography. | Hormonal disorders, immunological and hematological disorders, chromosomal and genetic anomalies, and renal failure. | 33.88±6.32 vs 33.13±5.00 | Not stated | 0.5 ml PRP was infused into uterine cavity 48h before embryo transfer | No intervention | Long agonist, or antagonist protocol | Frozen ET | Clinical pregnancy rate, ongoing pregnancy rate, miscarriage rate, multiple pregnancy rate, chemical pregnancy rate |

1. Baum M, Yerushalmi GM, Maman E, Kedem A, Machtinger R, Hourvitz A, et al. Does local injury to the endometrium before IVF cycle really affect treatment outcome? Results of a randomized placebo controlled trial. Gynecological endocrinology : the official journal of the International Society of Gynecological Endocrinology. 2012;28(12):933-6.

2. Gurgan T, Kalem Z, Kalem MN, Ruso H, Benkhalifa M, Makrigiannakis A. Systematic and standardized hysteroscopic endometrial injury for treatment of recurrent implantation failure. Reproductive biomedicine online. 2019;39(3):477-83.

3. Karimzadeh MA, Ayazi Rozbahani M, Tabibnejad N. Endometrial local injury improves the pregnancy rate among recurrent implantation failure patients undergoing in vitro fertilisation/intra cytoplasmic sperm injection: A randomised clinical trial. Australian and New Zealand Journal of Obstetrics and Gynaecology. 2009;49(6):677-80.

4. Shahrokh-Tehraninejad E, Dashti M, Hossein-Rashidi B, Azimi-Nekoo E, Haghollahi F, Kalantari V. A randomized trial to evaluate the effect of local endometrial injury on the clinical pregnancy rate of frozen embryo transfer cycles in patients with repeated implantation failure. Journal of Family and Reproductive Health. 2016;10(3):108-14.

5. Shohayeb A, El-Khayat W. Does a single endometrial biopsy regimen (S-EBR) improve ICSI outcome in patients with repeated implantation failure? A randomised controlled trial. European Journal of Obstetrics and Gynecology and Reproductive Biology. 2012;164(2):176-9.

6. Tang Z, Hong M, He F, Huang D, Dai Z, Xuan H, et al. Effect of endometrial injury during menstruation on clinical outcomes in frozen-thawed embryo transfer cycles: A randomized control trial. Journal of Obstetrics and Gynaecology Research. 2020;46(3):451-8.

7. Davari-Tanha F, Tehraninejad ES, Ghazi M, Shahraki Z. The role of G-CSF in recurrent implantation failure: A randomized double blind placebo control trial. International journal of reproductive biomedicine. 2016;14(12):737-42.

8. Eftekhar M, Miraj S, Mojtahedi MF, Neghab N. Efficacy of intrauterine infusion of granulocyte colony stimulating factor on patients with history of implantation failure: A randomized control trial. International journal of reproductive biomedicine. 2016;14(11):687-90.

9. Huang P, Yao C, Wei L, Lin Z. The intrauterine perfusion of granulocyte-colony stimulating factor (G-CSF) before frozen-thawed embryo transfer in patients with two or more implantation failures. Human Fertility. 2020.

10. Kalem Z, Namli Kalem M, Bakirarar B, Kent E, Makrigiannakis A, Gurgan T. Intrauterine G-CSF Administration in Recurrent Implantation Failure (RIF): An Rct. Scientific reports. 2020;10(1):5139.

11. Obidniak D, Gzgzyan A, Dzhemlikhanova L, Feoktistov A. Effect of colony-stimulating growth factor on outcome of frozen-thawed embryo transfer in patients with repeated implantation failure. Fertility and sterility. 2016;106 (Supplement 3):e134-e5.

12. Torky H, El-Desouky ES, El-Baz A, Aly R, El-Taher O, Shata A, et al. Effect of Intra Uterine Granulocyte Colony Stimulating Factor vs. Human Chorionic Gonadotropin at Ovum Pick Up Day on Pregnancy Rate in IVF/ICSI Cases With Recurrent Implantation Failure. JBRA assisted reproduction. 2021.

13. Huang P, Wei L, Li X. A study of intrauterine infusion of human chorionic gonadotropin (hCG) before frozen-thawed embryo transfer after two or more implantation failures. Gynecological Endocrinology. 2017;33(1):67-9.

14. Wang M, Deng H, Ye H. Intrauterine injection of human chorionic gonadotropin improves pregnancy outcome in patients with repeated implantation failure in frozen-thawed embryo transfer. [Chinese]. Zhong nan da xue xue bao. 2019;Yi xue ban = Journal of Central South University. Medical sciences. 44(11):1247-51.

15. Madkour A, Bouamoud N, Louanjli N, Kaarouch I, Copin H, Benkhalifa M, et al. Intrauterine insemination of cultured peripheral blood mononuclear cells prior to embryo transfer improves clinical outcome for patients with repeated implantation failures. Zygote (Cambridge, England). 2016;24(1):58-69.

16. Pourmoghadam Z, Soltani-Zangbar MS, Sheikhansari G, Azizi R, Eghbal-Fard S, Mohammadi H, et al. Intrauterine administration of autologous hCG- activated peripheral blood mononuclear cells improves pregnancy outcomes in patients with recurrent implantation failure; A double-blind, randomized control trial study. Journal of reproductive immunology. 2020;142 (no pagination)(103182).

17. Yu N, Zhang B, Xu M, Wang S, Liu R, Wu J, et al. Intrauterine administration of autologous peripheral blood mononuclear cells (PBMCs) activated by HCG improves the implantation and pregnancy rates in patients with repeated implantation failure: a prospective randomized study. American Journal of Reproductive Immunology. 2016;76(3):212-6.

18. Nazari L, Salehpour S, Hosseini MS, Hashemi Moghanjoughi P. The effects of autologous platelet-rich plasma in repeated implantation failure: a randomized controlled trial. Human Fertility. 2020;23(3):209-13.

19. Nazari L, Salehpour S, Hosseini S, Sheibani S, Hosseinirad H. The Effects of Autologous Platelet-Rich Plasma on Pregnancy Outcomes in Repeated Implantation Failure Patients Undergoing Frozen Embryo Transfer: A Randomized Controlled Trial. Reproductive sciences (Thousand Oaks, Calif). 2022;29(3):993-1000.

20. Safdarian L, Aleyasin A, Aghahoseini M, Lak P, Mosa SH, Sarvi F, et al. Efficacy of the Intrauterine Infusion of Platelet-Rich Plasma on Pregnancy Outcomes in Patients With Repeated Implantation Failure: A Randomized Control Trial. International Journal of Women's Health and Reproduction Sciences. 2022;Vol.10(1):38-44p.

21. Zamaniyan M, Peyvandi S, Heidaryan Gorji H, Moradi S, Jamal J, Yahya Poor Aghmashhadi F, et al. Effect of platelet-rich plasma on pregnancy outcomes in infertile women with recurrent implantation failure: a randomized controlled trial. Gynecological Endocrinology. 2021;37(2):141-5.
